# Editorial: Microbial Bioenergetics

**DOI:** 10.3389/fmicb.2021.793917

**Published:** 2021-11-22

**Authors:** Catarina M. Paquete, Wolfgang Nitschke, Fevzi Daldal, Davide Zannoni

**Affiliations:** ^1^Instituto de Tecnologia Química e Biológica António Xavier, Universidade Nova de Lisboa, Oeiras, Portugal; ^2^Aix Marseille Université, CNRS, BIP (UMR 7281), Marseille, France; ^3^Department of Biology, University of Pennsylvania, Philadelphia, PA, United States; ^4^Department of Pharmacy and Biotechnology, University of Bologna, Bologna, Italy

**Keywords:** bioenergetics, respiration, electron transfer, energy transduction systems, microorganism

In nature, microorganisms use different processes to harness energy from a variety of metabolic pathways, converting, or storing it into chemical or electrochemical forms. Some organisms are capable of acquiring energy from light through photosynthesis, whereas others break down the chemical bonds of organic compounds to release energy. The variety of environments in which microorganisms can thrive is enormous, ranging from extreme pH and ionic strengths, pressure, and temperature to conditions where oxygen is scarce or even absent. *Microbial bioenergetics* aims to understand how microorganisms efficiently coordinate their energy needs with their different cellular physiologies, including respiration, metabolism, storage, and other biological processes. The scope of this special issue on this Research Topic is to focus on recent advances of structural, functional, and molecular biological natures of energy transduction systems in microbes. It consists of 13 articles by several groups actively engaged on these topics, with 10 of them describing original research while the remaining are reviews and method description.

The review by Buckel covers energy conservation during fermentation in anaerobic bacteria focusing on electrochemical ion gradients for ATP synthesis. These processes can be catalyzed by ubiquitous H^+^-or Na^+^-dependent ATP-synthases, documenting that anaerobes can be more efficient than aerobes. In particular, comprehensive descriptions of the biotin-containing decarboxylases and NAD:ferredoxin oxidoreductases are provided in this review, and the mechanisms of the recently discovered electron bifurcation are examined in detail.

Respiration being a major process shaping the biology of many environments, Esposti et al. focuses on heme A containing cytochrome oxidases (COX), with the aim of elucidating the evolutionary origin of COX in bacteria. Toward this, the authors used extensive sequence and phylogenetic analyses to show that the ancestors of extant iron-oxidizers were the first species to evolve a COX, suggesting that the earliest geochemical evidence for bacterial respiration seems to originate from acidophilic iron-oxidizing prokaryotes.

Several organisms, including bacteria, archaea, and fungi perform efficient extracellular electron transfer processes. In their research article, Tanaka et al. described these processes for the photosynthetic cyanobacterium *Synechocystis* sp. PCC 6803. The authors investigate the capabilities of this species in reducing iron hydroxides and generating (photo)electricity under a variety of physiological conditions. Their study reveals that photosynthetic microorganisms can perform, in glucose-enriched environments, ferrihydrite reduction by extracellular electron transfer processes. Bertling et al. studied the aerobic respiration carried out by the facultative anaerobe *Shewanella oneidensis* MR-1, showing that in this species the *aa*_3_-type cytochrome *c* oxidase, which is the primary oxygen reductase in aerobiosis in many bacteria, does not appear to contribute to aerobic growth in *Shewanella*. Instead, this activity and its contribution to aerobic growth is dependent on the energy and carbon source that the bacterium uses. In addition, the authors also examine the role of cAMP-receptor protein CRP and phosphodiesterase CpdA in regulating respiration in *Shewanella*, demonstrating that CRP proteins are important for aerobic respiration. In respect to extracellular electron transfer, it is known that *Shewanella* transfers electrons outside of the cell using the outer membrane-associated MTR complex, composed of the decaheme cytochromes MtrA and MtrC together with the 26-strand beta-barrel protein MtrB. Earlier studies have shown that *in vitro* mixing of purified MtrAB with soluble MtrC results in spontaneous formation of a stable high-affinity complex. In their research article Piper et al. now demonstrate that the complex thus formed has a similar structure and rate of electron transfer as compared with the MTR complex directly purified from *S. oneidensis* MR-1.

*Leptospirillum* spp. are Gram-negative, obligately acidophilic chemoautotrophic prokaryotes that can respire aerobically using reduced soluble iron or ferrous iron-containing sulfide minerals. Blake et al. explore in details the respiratory chain components of these organisms, identifying the periplasmic cytochrome 579 as a possible candidate involved in this process. Their research article describes selected structural and functional properties of cytochrome 579 after its purification to electrophoretic homogeneity from cell-free extracts. The results obtained support the hypothesis that cytochrome 579 is a periplasmic electron conductor from the outer membrane associated initial iron oxidation to a terminal reductase in the plasma membrane of *Leptospirillum*.

Copper (Cu) is a micronutrient that is an essential cofactor in numerous redox enzymes in all domains of life, but it can also be deleterious to cells in excess. Thus, cells have developed sophisticated Cu homeostasis processes to efficiently provide Cu to cuproproteins while controlling its toxicity. The work of Öztürk et al. investigates the maturation of an extracytoplasmic multicopper oxidase CutO from the Gram-negative facultative phototroph *Rhodobacter capsulatus*, involved in periplasmic Cu detoxification. The laccase-like multicopper oxidases are unique because Cu is both a substrate and a cofactor for these enzymes, yet how they acquire Cu essential for their activities remain unknown. In this research article, the authors investigated the cellular source of Cu for CutO assembly and found that it is partly provided by Cu detoxification pathway centered around the Cu-transporting P_1B_-type ATPase, CopA1. Moreover, they showed that a small protein CutF, clustered with *cutO* structural gene on some bacterial genomes, is required for maturation of CutO activity. Bacterial species often contain multiple P_1B_P1-type ATPases, with CopA1-type dedicated to cytoplasmic detoxification and CopA2-type providing Cu to some cuproenzymes. The heme-copper oxidases of *aa*_3_- or *cbb*_3_-types are proton-translocating respiratory cytochrome oxidases that contain a binuclear heme-copper site where oxygen reduction occurs. In a related work, Andrei et al. investigated the CopA_2_-type P_1B_-type ATPases, and showed that, as predicted, they provide Cu for maturation of the *cbb*_3_-type cytochrome oxidases. By reconstituting the purified membrane-integral CopA_2_-type ATPase CcoI from *R. capsulatus* into liposomes and monitoring Cu transport by solid-supported membrane electrophysiology, the authors demonstrate that CcoI provides the link between the cytosolic Cu and the periplasmic Cu chaperones networks during the maturation of *cbb*_3_-type cytochrome oxidase. Remarkably, the pathogenic bacterium *Campylobacter jejuni* is among the few bacterial species that contain only a *cbb*_3_-type cytochrome oxidase. Garg et al. identified the genes involved in the assembly, and activity, of this type cytochrome oxidase, and used mutagenesis to confirm their involvements in these processes. Most, not all, of these genes are highly homologous to their counterparts in other species, linking copper homeostasis, and cytochrome oxidase maturation in this pathogen.

Respiratory processes also play important roles in mycobacterial growth and survival as well as their response and adaptation to different environmental niche and stress, often leading to selection of “persisters” cell population in infected patients. Patil and Jain investigated in detail the mechanisms involved in transitioning to an energy-compromised state in this species upon deletion of the *atp*D gene, revealing that a tight relationship between energy metabolism, redox homeostasis, and lipid biosynthesis exist during ATP-depleted states of mycobacterial cells. This information may be crucial to identify and develop novel therapeutic drug targets to counter mycobacterial infections.

The review article by Gorka et al. surveys the structures and functions of primary electron donors in Type I- and Type II-photosynthetic reaction centers using the vast body of spectroscopic research that has been performed to date. In this review, density functional theory calculations on each oxidized primary donor are used to analyse the electronic properties of the involved electron donors, and the properties thus defined are used to compare and contrast the hetero- and homodimeric photosynthetic reaction centers.

In order to investigate the role of microbial rhodopsin in the heterotrophic growth via phototrophy by *Actinobacteria*, Chuon et al. isolated and characterized the actinorhodopsin ActR-13023 from *Candidatus Aquiluna* sp. IMCC13023. Their research article demonstrated that purified ActR-13023 could be reconstituted with actinobacterial carotenoids for additional light-harvesting, confirming the functional role of this gene and its product, ActR. The role of ActR for solar energy capture by microbial communities is also highlighted in this study. Lastly, Cypionka and Reese contribute a methods article, which describes a freeware app, proton.exe that can be used to assess metabolic activities associated with proton uptake/release processes based on Michaelis-Menten kinetics or first order kinetics. *Proton.exe* models ATP gain and proton-motive force changes in cells, allowing researchers to study proton-related metabolism in mitochondria, phototrophic bacteria, and chloroplasts. The authors used *Desulfovibrio desulfuricans* CNS to validate their model for different biological processes: sulfate uptake by proton-sulfate symport, scalar alkalinization by sulfate reduction to sulfide, nitrate, and nitrite reduction to ammonia as well as electron transport-coupled proton translocation.

Overall, this special issue greatly contributes to the current knowledge of various *microbial bioenergetics* subfields and provides the readers with a wealth of mostly current information to extend their understanding and appreciation of these vibrant and ever developing areas of the microbial world.

## Author Contributions

CP wrote the first draft of the manuscript. CP, WN, FD, and DZ contributed to the final version of the manuscript. All authors contributed to the article and approved the submitted version.

## Conflict of Interest

The authors declare that the research was conducted in the absence of any commercial or financial relationships that could be construed as a potential conflict of interest.

## Publisher's Note

All claims expressed in this article are solely those of the authors and do not necessarily represent those of their affiliated organizations, or those of the publisher, the editors and the reviewers. Any product that may be evaluated in this article, or claim that may be made by its manufacturer, is not guaranteed or endorsed by the publisher.

